# Prevalence of Focal Inner, Middle, and Combined Retinal Thinning in Diabetic Patients and Its Relationship With Systemic and Ocular Parameters

**DOI:** 10.1167/tvst.10.2.26

**Published:** 2021-02-17

**Authors:** Rony Carlos Preti, Claudio Iovino, Maria Fernanda Abalem, Rafael Garcia, Helen Nazareth Veloso dos Santos, Gustavo Sakuno, Adrian Au, Leonardo Provetti Cunha, Leandro Cabral Zacharias, Mario Luiz Ribeiro Monteiro, Srinivas Reddy Sadda, David Sarraf

**Affiliations:** 1 Division of Ophthalmology, University of São Paulo Medical School, São Paulo, Brazil; 2 Eye Clinic, Multidisciplinary Department of Medical, Surgical and Dental Sciences, University of Campania “Luigi Vanvitelli,” Naples, Italy; 3 Kellogg Eye Center, University of Michigan, Ann Arbor, MI, USA; 4 Stein Eye Institute, David Geffen School of Medicine at UCLA, Los Angeles, CA, USA; 5 Department of Ophthalmology, School of Medicine, Federal University of Juiz de Fora, Juiz de Fora, Brazil; 6 Doheny Eye Institute, Los Angeles, CA, USA; 7 Greater Los Angeles Veterans Affairs Healthcare Center, Los Angeles, CA, USA

**Keywords:** superficial capillary plexus ischemia, deep capillary plexus ischemia, diabetic retinopathy, optical coherence tomography, retinal thinning

## Abstract

**Purpose:**

To determine the prevalence of focal inner, middle, and combined inner/middle retinal thinning (FIRT, FMRT, and FCRT, respectively) in different stages of diabetic retinopathy (DR) without diabetic macular edema and to assess the relationship between such findings with ocular and systemic parameters.

**Methods:**

This was a cross-sectional, comparative study comprising healthy participants and diabetic patients with different stages of DR. Forty-nine horizontal macular B-scans from the selected eye were obtained using spectral-domain optical coherence tomography (SD-OCT) and analyzed for the presence of FIRT, FMRT, or FCRT and any relationship with systemic and ocular parameters. Focal retinal thinning (FRT) was subjectively defined as any evidence of inner and/or middle retinal thinning.

**Results:**

A total of 190 participants (52 healthy participants and 138 diabetic patients) were included. A higher prevalence of FRT was observed in eyes with advanced DR versus healthy eyes and versus diabetic eyes with no DR or mild DR. FIRT and FCRT were significantly greater in eyes with proliferative DR treated with pan-retinal photocoagulation, and FMRT was significantly more common in eyes with severe nonproliferative DR. FRT was significantly more common in patients with coronary artery disease and was positively correlated with diabetes duration, serum creatinine, and glycosylated hemoglobin and negatively correlated with age, estimated glomerular filtration rate, and visual acuity.

**Conclusions:**

FRT occurs in all stages of DR and is increasingly prevalent with increasing severity of DR.

**Translational Relevance:**

OCT identification of FRT may provide a surrogate biomarker of retinal and systemic disease in diabetic patients.

## Introduction

Diabetic retinopathy (DR), one of the leading causes of blindness in the world, may be the result of various pathogenic mechanisms, including neurodegeneration and increased oxidative stress, release of proinflammatory mediators, platelet aggregation, leukocyte activation and adherence to the vessel wall, and glutamate excitotoxicity.^1^^–^^3^ All the aforementioned factors lead to a disruption of the blood-retinal barrier and death of vascular endothelial cells.^4^ As a consequence, tissue perfusion is compromised, causing ischemia at various levels of severity that lead to the production of vascular endothelial growth factor (VEGF).^5^

Fluorescein angiography (FA) has been utilized for more than 50 years to study the retinal microvasculature, producing a wealth of knowledge about normal and diseased retina.^6^ However, only the superficial retinal capillary plexus (SCP) can be sufficiently resolved using this ancillary imaging modality, and abnormalities in the intermediate (ICP) and deep retinal capillary plexus (DCP) are not optimally displayed.^7^

While the SCP provides the main blood supply to the retinal nerve fiber layer (RNFL) and the ganglion cell layer (GCL), the ICP and DCP nourish the inner plexiform layer (IPL), the inner nuclear layer (INL), and the outer plexiform layer (OPL).^8^^,^^9^ Disturbance of blood flow in any of these retinal plexuses can lead to ischemia. SCP hypoperfusion clinically manifests in the acute phase as a cotton-wool spot (CWS),^10^ a focal area of thickening and hyperreflectivity of the inner retinal layers with spectral-domain optical coherence tomography (SD-OCT).^11^ Hypoperfusion of the DCP causes infarction of the INL and is acutely illustrated as a band of INL hyperreflectivity with SD-OCT referred to as paracentral acute middle maculopathy (PAMM).^12^ Both CWS and PAMM lesions leave a legacy of thinning of the inner and middle retina, respectively, due to infarction and cell death.^12^^,^^13^

Yu et al.^13^ in 2014, using multimodal imaging, described the presence of either focal inner retinal thinning (FIRT) or focal middle retinal thinning (FMRT) in diabetic patients and attributed these pathoanatomic findings to SCP and DCP nonperfusion, respectively, and demonstrated that both abnormalities impair visual function. However, the prevalence of such findings in the diabetic population, and its relationship with the severity of DR, is still unknown.

The purpose of this study was to ascertain the prevalence of FIRT, FMRT, and focal combined retinal thinning (FCRT) in different stages of DR using SD-OCT and to correlate these measurements with the severity of systemic and ocular parameters.

## Methods

This observational, cross-sectional, consecutive, masked, and comparative study was performed in accordance with the principles of the Declaration of Helsinki and was approved by the Institutional Review Board Ethics Committee at the University of São Paulo Medical School, São Paulo, Brazil. Informed consent was obtained from all patients prior to enrollment.

Type 1 and 2 diabetic patients without diabetic macular edema (DME) and without disorganized retinal inner layers (DRIL)^14^ with best-corrected visual acuity (BCVA) ≥20/200 were included. The exclusion criteria were history of macular grid laser, pars plana vitrectomy, intravitreal injections (either steroids or anti-VEGF), vitreomacular traction, and evidence of significant media opacity (e.g., cataract or vitreous hemorrhage) or any other ocular disorder that could potentially impair OCT evaluation.

From the total cohort, only the eye with the better BCVA was chosen for the analysis, and for participants with the same BCVA in both eyes, the study eye was randomly selected.

### Systemic Evaluation

Patients self-reported their age, gender, race, type and duration of diabetes, and presence of any systemic disease, including systemic arterial hypertension (SAH), dyslipidemia, and coronary artery disease (CAD). Deidentified medical records were evaluated, and all medications and medical and surgical history were ascertained. Blood pressure, weight, and height were then measured and body mass index (BMI) was calculated. A blood test was requested for each patient to measure fasting plasma glucose (FPG), glycosylated hemoglobin (A1C), and serum creatinine at baseline enrollment. Estimated glomerular filtration rate (eGFR) was calculated from serum creatinine.^15^ Capillary plasma glucose (CPG) was performed at each enrollment visit.

### Ocular Clinical Examination

All participants underwent a comprehensive ophthalmologic evaluation that included clinical history, BCVA using the Snellen chart at 4 m further converted to logMAR, anterior segment slit-lamp examination, intraocular pressure measured by Goldmann applanation tonometry, and dilated biomicroscopic retinal examination with a 78-diopter lens. Ancillary testing included SD-OCT (Spectralis; Heidelberg Engineering, Heidelberg, Germany) and ocular biometry (IOL Master 500; Carl Zeiss Meditec AG, Jena, Germany) for axial length.

Patients were classified by DR severity on fundus examination (i.e., mild, moderate, and severe nonproliferative DR or NPDR and PDR) according to the International Clinical Disease Severity Scale.^16^ A PDR group with pan-retinal photocoagulation (PRP) scars but without DME was also included.

### OCT Analysis of the Retina and Choroid

A 20° × 20° volume scan containing 49 horizontal B-scans in enhanced-depth imaging mode was obtained for all study eyes. All measurements were performed with the Heidelberg Eye Explorer (version 1.8.6.0) using the HRA/Spectralis Viewing Module (version 5.8.3.0).

Central subfield thickness (CST) was calculated by the built-in automated software, which measures the distance between the internal limiting membrane and the retinal pigment epithelium. The CST was then obtained from the 1000-µm diameter Early Treatment Diabetic Retinopathy Study inner-circle grid map placed over the macula. If an eye displayed any intraretinal cysts and/or a CST value greater than 320 µm for males and 305 µm for females,^17^ a diagnosis of DME was rendered and the eye was excluded from the analysis.

**Figure 1.**
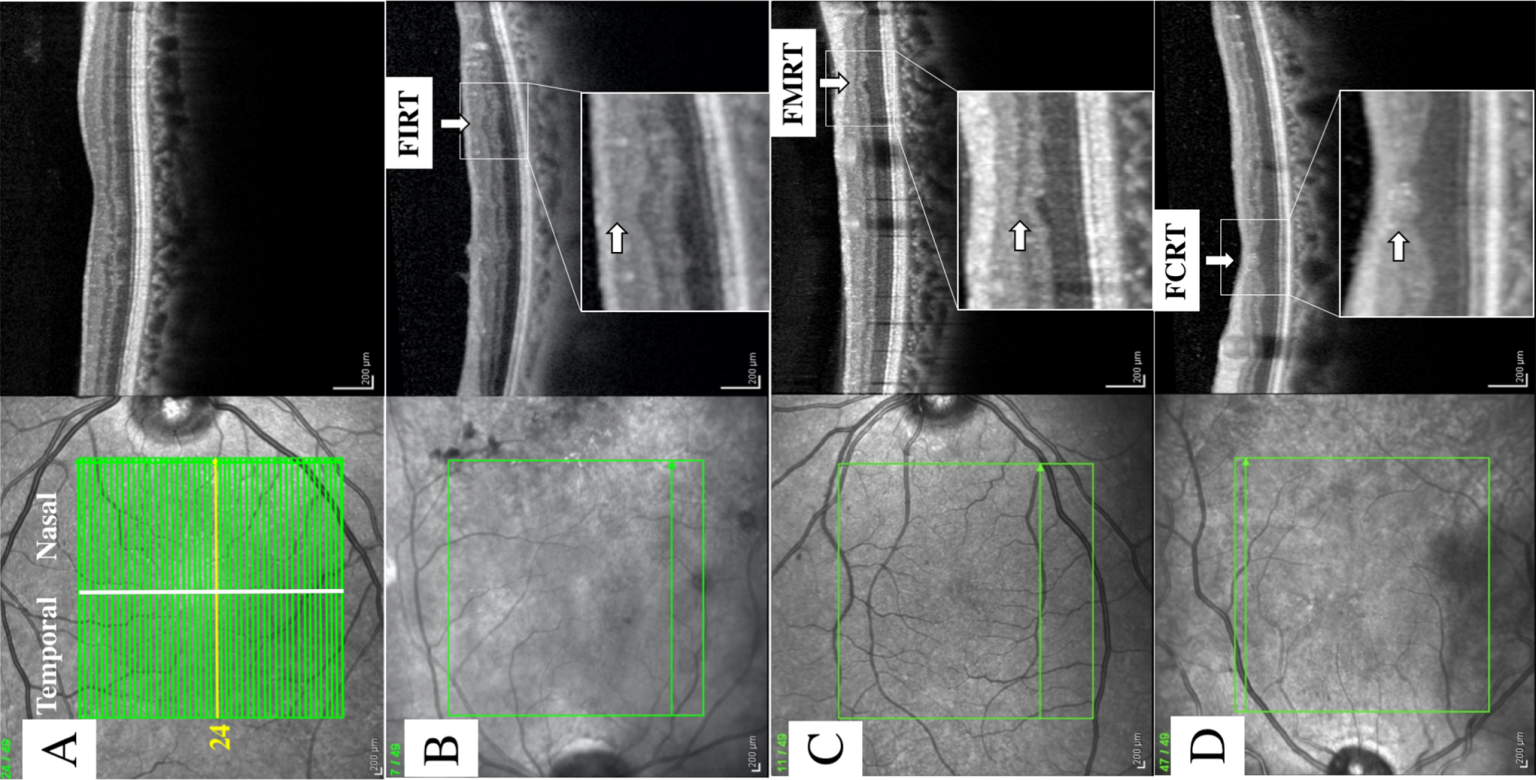
(A) Right eye of a 70-year-old male diabetic patient illustrates the 49 temporal and nasal B-scans used to calculate FIRT, FMRT, and FCRT, respectively. (B) An example of FIRT in the temporal macula of the left eye of a 52-year-old female patient with proliferative DR is shown. (C) An example of FMRT in the nasal macula of the right eye of a 55-year-old male patient with mild DR is displayed. (D) An example of FCRT in the nasal macula of the left eye of a 59-year-old female patient with severe DR is illustrated.

Each of the 49 horizontal macular B-scans was divided into nasal and temporal sectors (Fig. 1A) and evaluated by two masked investigators (RCP and MFA) for the presence of FIRT, FMRT, or FCRT. Specifically, focal thinning of the RNFL and GCL was recorded as FIRT (Fig. 1B). The presence of focal thinning of the IPL, INL, and OPL was classified as FMRT (Fig. 1C). The presence of both FIRT and FMRT in the same B-scan position was recorded as FCRT (Fig. 1D). Focal retinal thinning (FRT) was deemed to be present if any of these three gradings (FIRT, FMRT, FCRT) were identified (Figs. 1B–D). The retinal position below major retinal vessels was excluded from this analysis due to confounding effect of physiologic retinal thinning.

The dense volume scan was divided into temporal and nasal sectors by tracing a vertical line passing through the central foveal depression to determine if thinning was more prevalent in either sector (Fig. 1A). A binary system of quantitation was used to record the presence, recorded as 1 point, or absence, recorded as 0, of FIRT, FMRT, and FCRT in each of the 49 B-scans in the temporal and/or nasal retinal sectors. In order to assess the impact of FRT on VA, the location of FRT (foveal versus extrafoveal), as well as the disruption of the ellipsoid zone (EZ) and interdigitation zone (IZ) in the foveal region, defined as focal discontinuity or impairment of the EZ/IZ zone band on SD-OCT, was determined. In a single B-scan, in each macular sector, nasal or temporal, a maximum of 1 point could be registered for each type of retinal thinning (FIRT, FMRT, and FCRT). If all three forms of retinal thinning were present within a given temporal or nasal B-scan, then a maximum of 1 point would be registered for each. The maximum score that a case could accumulate for each of the three categories (FIRT, FMRT, FCRT) was therefore 147 (3 × 49) for the nasal and 147 (3 × 49) for the temporal sector, respectively. A total aggregated score (TAS) of focal retinal thinning was assigned for a maximum point score of 294 (147 + 147) that included both the temporal and nasal sectors of the entire volume scan for a given case. Intergrader analysis was performed to compare the scores of the two masked readers.

### Correlation Analysis

Correlation of FIRT, FMRT, and FCRT versus systemic parameters (i.e., gender, race, diabetes mellitus [DM] type, DR stage, and presence of SAH, dyslipidemia, and CAD), versus quantitative factors (i.e., age, A1C level, BMI, systolic and diastolic blood pressure), and versus ocular parameters (BCVA and CST) was performed.

### Statistical Analysis

All analyses were performed with the software IBM SPSS Statistics, version 15.0 (SPSS, Inc, Chicago, IL, USA) or Stata 11.0 (StataCorp, College Station, TX, USA). The collected data were analyzed using descriptive statistics. Shapiro-Wilk's *W* test and graphical analysis were used to check for normal distribution. The intraclass correlation coefficient statistic was used to test intergrader reliability. One-way analysis of variance was used to determine difference in means between TAS and FRT (FIRT, FMRT, and FCRT) with DR stages. Post hoc Tukey honestly significant difference (HSD) was used to confirm statistically significant comparisons. Pearson correlation coefficient was used to determine any correlation between FRT and systemic *quantitative* variables with *P* values adjusted with Bonferroni correction. Linear regression was performed between TAS of FRT with highly correlative variables. Student's *t*-test was used to compare FRT between patients with and without systemic variables (e.g., presence absence of CAD or DR). *P* < 0.05 was considered statistically significant unless otherwise stated.

## Results

A total of 245 participants (186 with DM and 59 healthy participants) were initially screened and 190 (138 diabetic patients and 52 healthy participants) met the inclusion criteria. The right eye was selected in 135 patients (71%) in the study. Mean ± SD age at presentation was 59 (14) years in diabetic patients and 58 (14) years in healthy participants, and the difference was not statistically significant (*P* = 0.67). Demographic, systemic, and ocular features of all study participants are summarized in Table 1. Of the 138 diabetic patients, 70 (48.3%) exhibited no signs of DR, while 24 (16.6%) displayed mild, 19 (13.1%) moderate, and 6 (4.1%) severe, nonproliferative DR, and 5 (3.4%) displayed PDR and 14 (9.7%) manifested PDR treated with PRP. In comparison to controls, patients with diabetes exhibited a statistically significant higher FPG, CPG, A1C, serum creatinine, systolic blood pressure, and BMI (Table 1).

**Table 1.** Baseline Characteristics of Control Participants Versus Diabetic Patients

Characteristic	Controls	Diabetes	*P* Value
No. of patients	52	138	
Age,^a^ mean (SD), y	58 (14)	59 (14)	0.67
Sex: men, No. (%)	32 (58)	77 (56)	0.87
DM type, No. (%)			
Type 1	0	18 (12.4)	
Type 2	0	120 (82.8)	
FPG, mean (SD)	93 (19)	156 (76)	<0.001
CPG, mean (SD)	90 (44)	162 (80)	<0.001
A1C, mean (SD), %	5.5 (0.8)	8 (1.7)	<0.001
DM, duration, y, mean (SD) (No., % ≤10 years)	0	12.6 (8.3) (76, 52.4)	<0.001
Presence of SAH in DM patients, No. (%)	7 (14.6)	110 (80)	<0.001
eGFR,^a^ mean (SD)	102 (13.5)	80.4 (34)	0.087
Presence of dyslipidemia, No. (%)	3 (6)	17 (12)	0.29
Serum creatinine, mean (SD)	0.76 (0.15)	1.28 (1.3)	<0.001
Blood pressure			
Systolic blood pressure, mean (SD), mm Hg	124.75 (11)	135.31 (22)	<0.001
Diastolic blood pressure, mean (SD), mm Hg	82.3 (7.8)	81.13 (11.1)	0.50
Body mass index, mean (SD)	25.5 (2.7)	28.7 (5.5)	<0.001
Presence of CAD, No. (%)	0	4 (3)	0.57
Ophthalmic measurements			
Visual acuity, mean (SD), logMAR	0.01 (0.06)	0.13(0.2)	<0.001
Axial length, mean (SD), µm	22.10 (98)	23.12 (0.8)	<0.001
DR classification, No. (%)			
DM without DR		70 (48.3)	
Mild NPDR		24 (16.6)	
Moderate NPDR		19 (13.1)	
Severe NPDR		6 (4.1)	
PDR		5 (3.4)	
PDR and PRP		14 (9.7)	

a No age statistical significant difference was observed between healthy participants and different stages of DR.

### OCT Analysis of the Retina

**Figure 2.**
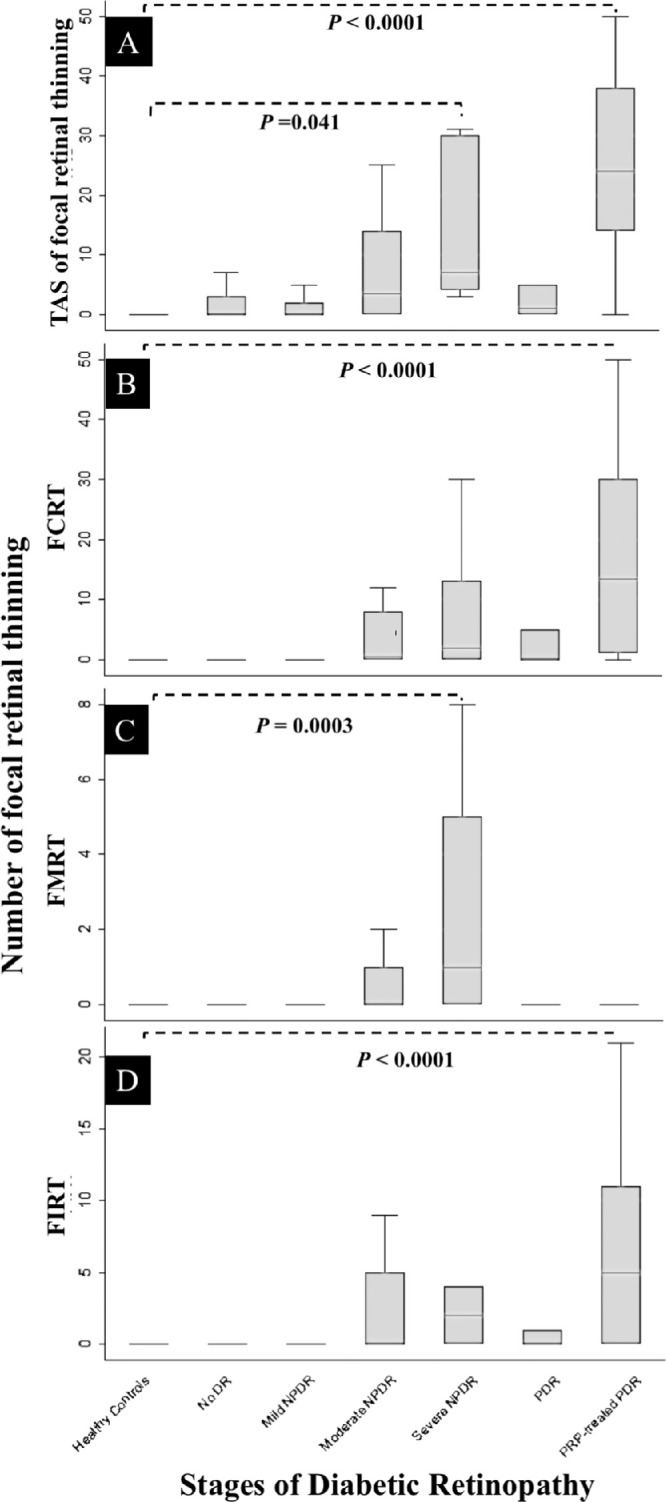
Boxplot of mean values for TAS of focal retinal thinning, FCRT, FMRT, and FIRT (A, B, C, and D, respectively) plotted against healthy controls and grade of DR.

The mean (SD) CSTs of the control and diabetic groups were 280 (17) and 274 (39) µm, respectively (*P* = 0.15). Table 2 and Figure 2 display the mean scores for TAS and for all categories of FRT (i.e., FIRT, FMRT, FCRT), as well as the significant *P* values for the comparisons of the various FRT categories between the DR groups and the normal controls.

**Table 2.** Comparison of Mean Values for TAS and for Each Subcategory of FRT in Healthy Participants versus Diabetic Patients with Different Stages of DR

Patient	DR Stage	No. of Eyes	Mean TAS of FRT	SD	*P* Value^a^
Healthy participants		52	0.8	3.4	
	DM without DR	70	2.8	7.6	
	Mild NPDR	24	2.3	4.4	
	Moderate NPDR	19	10.9	19.8	
	Severe NPDR	6	13.7	13.2	0.041
	PDR	5	8	14.7	
	PDR and PRP	14	32.1	30.1	<0.001
	Mean Number of FRT Category in Each DR Stage				
Patient	DR Stage	No. of Eyes	FIRT, Mean (SD)	FMRT, Mean (SD)	FCRT, Mean (SD)

Healthy participant		52	0.20 (0.9)	0.13 (0.7)	0.4 (2.5)
	DM without DR	70	1.6 (4.8)	0.3 (0.8)	0.8 (2.9)
	Mild NPDR	24	0.6 (1.2)	0.8 (2.6)	0.9 (2.7)
	Moderate NPDR	19	2.6 (3.9)	1.1 (1.9)	7.2 (19.2)
	Severe NPDR	6	3.4 (4.5)	2.5 (3.3)^a^	7.8 (11.9)
	PDR	5	1.4 (2.6)	0.5 (1.3)	6.4 (11.7)
	PDR and PRP	14	6.5 (6.6)^a^	0.5 (1.5)	25.1 (34.)^a^

a *P* < 0.05 with Bonferroni correction comparing FRT of diabetic patients with healthy participants.

All mean TAS values were greater in the diabetic cohort versus the normal control cohort, although this reached statistical significance only with the severe NPDR cohort and the PDR with PRP cohort (Fig. 2A, Table 2). Mean FRT (i.e., FIRT, FMRT, FCRT) scores increased in the groups with moderate nonproliferative DR stage (Figs. 2B–D and Table 2) and worse.

Within the diabetic cohort, post hoc Tukey HSD analysis showed that the PDR PRP group exhibited a statistically significantly greater FIRT mean score versus the group with no DR (*P* < 0.001) and versus the group with mild NPDR (*P* < 0.001). The mean FMRT score was statistically significantly greater in the severe NPDR cohort versus the diabetic cohort without DR (*P* = 0.003) and versus the mild NPDR cohort (*P* = 0.007). The mean FCRT score in the PDR with PRP group was statistically significantly greater versus all the other groups with less severe stages (*P* < 0.03).

Among 190 eyes, 9 (5%) were remarkable for disruption of EZ/IZ zone in the foveal region, and the mean ± SD logMAR VA was 0.38 ± 0.31 (20/50 ± 20/30). The group of eyes showing foveal EZ/IZ zone disruption was associated with worse VA, Mann-Whitney *U* test, *P* < 0.001.

From the total sample, 50 (26 %) and 28 (15%) eyes showed evidence of foveal and extrafoveal FRT, respectively. After excluding eyes with EZ/IZ disruption, 41 eyes (22%) and 28 eyes (15%) displayed foveal and extrafoveal FRT, respectively. Mean ± SD BCVA for the group of eyes without FRT versus those with extrafoveal and foveal FRT was 0.05 ± 0.16 (20/20 ± 20/25), 0.0 ± 0 (20/20 ± 20/20), and 0.18 ± 0.18 (20/32 ± 20/32), respectively. As expected, the group of eyes with foveal FRT exhibited a statistically significant worse BCVA when compared to the group without FRT and also compared to the group with extrafoveal FRT, *P* < 0.001 (Tukey HSD test). No statistically significant difference was observed between the group of eyes without FRT versus with extrafoveal FRT, *P* = 0.07 (Tukey HSD test). This result was further confirmed by the Kruskal-Wallis test, *P* < 0.0001.

Intergrader analysis was performed to evaluate consistency between graders for nasal, temporal, and TAS of FRT measurements. Coefficients (95% confidence interval) between the two graders for the nasal, temporal, and TAS of FRT measurements were 0.998 (0.995–0.999), 0.997 (0.994–0.999), and 0.998 (0.995–0.999), respectively.

### Correlation of FRT and Nominal Systemic Parameters

To determine if nominal systemic parameters affected FRT, we subgrouped patients according to gender, race, DM type, presence of DR, and history of SAH and CAD and compared these to our FRT categories. Among these nominal systemic parameters, presence of DR and history of CAD demonstrated a statistically significant difference in FRT (*P* < 0.0001 and *P* < 0.02, respectively). Specifically, patients with CAD and DR exhibited a higher FRT score than those without CAD and without DR. Among FRT categories, only FIRT demonstrated a statistically significant association with presence of DR and CAD, *P* < 0.002 and *P* = 0.005, respectively. There was no statistically significant association between FRT and gender, race, and type of DM (*P* > 0.05).

### Correlation of FRT and Interval Systemic Parameters

Table 3 summarizes the correlations noted between interval systemic parameters versus FIRT, FMRT, FCRT, temporal TAS, nasal TAS, and composite TAS. Utilizing the conservative Bonferroni correction, DM duration and serum creatinine level were positively correlated with all categories of focal retinal thinning while A1C was only significantly correlated with FIRC and composite TAS. eGFR was negatively correlated with all categories of FRT, except FIRC.

**Table 3.** Correlations Between Each Category of FRT and TAS With Systemic Quantitative Parameters

Characteristic	Age	DM Duration	A1C	eGFR	Serum Creatinine	BMI	SBP	DBP
FIRT	−0.0777	0.1573^a^	0.3511^a^	−0.1277	0.1768^a^	0.1300	0.0164	−0.0115
*P* value	0.2856	0.0302	<0.0001	0.0831	0.0160	0.0787	0.8254	0.8767
FMRT	−0.0317	0.2301^a^	0.0685	−0.2086^a^	0.3032^a^	−0.1019	0.0907	0.0259
*P* value	0.6630	0.0014	0.3529	0.0044	0.0000	0.1688	0.2220	0.7280
FCRT	−0.1596^a^	0.1462^a^	0.0863	−0.2301^a^	0.2940^a^	−0.0389	0.0685	−0.1189
*P* value	0.0274	0.0441	0.2414	0.0016	<0.0001	0.5997	0.3569	0.1090
Temporal TAS	−0.1710^a^	0.2271^a^	0.2143^a^	−0.2481^a^	0.3024^a^	−0.0089	0.0876	−0.0846
*P* value	0.0180	0.0016	0.0033	0.0007	<0.0001	0.9041	0.2386	0.2548
Nasal TAS	−0.1481^a^	0.1466^a^	0.1332	−0.2641^a^	0.3721^a^	−0.0073	0.0547	−0.1268
*P* value	0.0409	0.0435	0.0699	0.0003	<0.0001	0.9218	0.4617	0.0873
TAS	−0.1700^a^	0.2013^a^	0.1863^a^	−0.2669^a^	0.3488^a^	−0.0086	0.0763	−0.1082
*P* value	0.0187	0.0053	0.0109	0.0002	<0.0001	0.9079	0.3047	0.1450

DBP, diastolic blood pressure; SBP, systolic blood pressure.

a *P* < 0.05 with Bonferroni correction.

**Figure 3.**
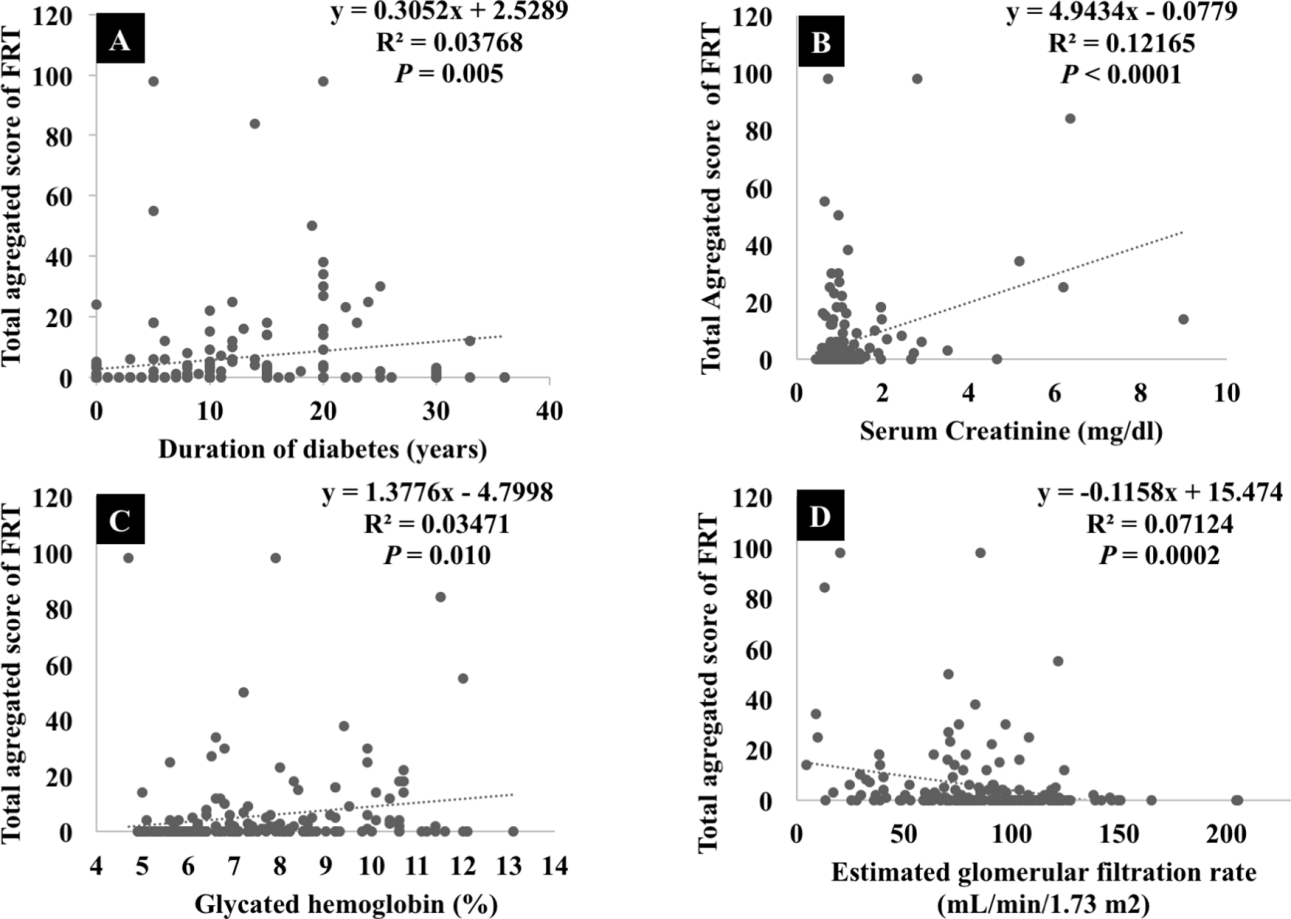
Scatterplot with linear regression of TAS of focal retinal thinning plotted against (A) duration of diabetes, (B) glycosylated hemoglobin, (C) eGFR, and (D) serum creatinine.

Figure 3 further confirms these correlations with a linear regression analysis of TAS with duration of diabetes, serum creatinine, A1C, and eGFR. Linear regression analysis indicates a positive correlation between duration and diabetes (Fig. 3A, *P* = 0.005), serum creatinine (Fig. 3B, *P* < 0.0001), A1C (Fig. 3C, *P* = 0.001), and TAS score (Fig. 3A, *P* = 0.0002). There was a negative correlation between eGFR and TAS score (Fig. 3D).

### Correlation Between FRT and Interval Ocular Parameters

When correlating the TAS and FRT categories (FIRT, FMRT, and FCRT) with VA, FCRT and TAS showed a statistically significant positive correlation utilizing the conservative Bonferroni correction (*r* = 0.34, *P* = 0.0001 and *r* = 0.31, *P* = 0.0004, respectively). The linear regression equation confirmed the positive correlation between TAS of FRT (*P* = 0.0004). This indicates that higher FRCT or TAS was associated with worse vision. No statistically significant different correlation was observed between FRT and macular CST (*P* > 0.05).

## Discussion

SD-OCT has become the dominant imaging tool for evaluation of the retina because of its many important features, including fast and easy image acquisition and high resolution and depth resolved capability. This study employed OCT to assess for FRT in the inner and middle retinal layers. Quantitative tools such as central macular thickness or even layer segmentation may detect only gross changes in retinal thickness and may not be effective in identifying localized alterations in the various retinal layers. The detection of these focal areas of thinning requires a meticulous methodical approach.

This study analyzed OCT volumes scans from a large data set of diabetic patients and healthy participants and searched for focal areas of retinal thinning in the inner and middle layers of the retina and found that FRT was significantly correlated with the stage of DR. In fact, the total aggregated score or TAS, which is a composite score of all the forms of FRT (FIRT, FMRT, FCRT), was greater at all stages of DR versus normal controls, including in the group without DR, although this difference was statistically significant in only the severe NPDR and PDR with PRP cohorts. Further statistical comparisons showed that the more advanced grades of retinopathy were associated with significantly higher scores of FRT versus those eyes with mild NPDR or no DR.

The significance of these OCT outcomes is further magnified by the systemic correlations. Mean TAS was positively correlated with duration of diabetes, hemoglobin A1C level, and serum creatinine level and negatively correlated with eGFR with statistical significance with all comparisons. The significance of such correlations is remarkable and indicates that FRT, detected with qualitative analysis of an OCT volume data set, may provide a surrogate biomarker of severity of retinal and even systemic disease. Development of OCT algorithms to detect focal inner and/or middle retinal thinning may have a great impact on the retinal and systemic care of diabetic patients and may have value for reading centers and clinical research trials.

The cause of inner and middle retinal thinning is unclear and may be the result of neuropathic or vasculopathic mechanisms. Sohn et al.^18^ found significant and progressive loss of the NFL and GCL by OCT in 45 patients without DR or with minimal DR and normal retinal capillary density with OCT angiography. Their results suggest that neuropathic injury may precede clinical and morphometric vascular changes caused by DM in some cases.^18^ However, various OCTA studies have noted the development of microvascular alterations in the SCP and DCP in the absence of clinical evidence of DR and have closely correlated the reduction in the microvascular density of the retinal capillary plexus with the severity of the stage of DR.^19^^–^^21^ Focal retinal thinning observed in this study of diabetic patients is likely a chronic alteration and the result of direct localized capillary injury to the SCP and/or the DCP. This is in contrast to PAMM or paracentral acute middle maculopathy lesions, which are the result of acute infarcts of the middle retina (i.e., the inner nuclear layer) due to hypoperfusion through the deep vascular plexus typically occurring in eyes with retinal artery or vein obstruction.^12^^,^^22^^,^^23^ While it is possible that some of the zones of FRT analyzed in this study may have resulted from prior PAMM lesions, most are likely due to direct capillary injury as typically occurs in DR.

The disorganization of the inner retinal structure on OCT, known as disorganization of inner retinal layers (DRIL), has been correlated with macular ischemia in patients with diabetes.^24^ Notably, DRIL was found to be associated with increasing DR severity and worsening VA in eyes with center-involved DME.^25^ The authors also reported an association with the disruption of outer retina layers and with epiretinal membrane formation.^25^ DRIL, however, represents a biomarker of advanced retinal disease while FRT may be even detected in eyes without DR and may provide a surrogate of systemic disease in patients with or without advanced DR. Moreover, FRT may represent a more direct consequence of retinal capillary injury and ischemia.

It is interesting that the TAS was negatively correlated with visual function. FRT localized in the fovea was most significantly associated with poor vision outcomes. It has been demonstrated that flow impairment within the DCP can lead to FRT with subsequent visual dysfunction.^13^ Dupas et al.^26^ also found a significant reduction of VA associated with capillary loss in the DCP evaluated with OCT angiography. However, some authors have demonstrated a weak correlation between macular ischemia and VA using OCTA^27^ and FA.^28^ Usui et al.^29^ proposed that VA may be affected by DCP ischemia due to the involvement of amacrine and horizontal cells that establish a highly interdependent neurovascular unit with capillaries in the ICP and DCP. The authors reported that damage to one or both of these components interferes with photoreceptor survival and function.

One of the main limitations of our study relates to the subjective identification of FRT and the nature of the grading system, which has not been previously validated. In order to reduce this form of bias, macular B-scans were evaluated by two masked investigators, and a high intergrader agreement was found. Another limitation is the relatively small numbers of participants in some of the subgroups. The group of patients with a history of CAD numbered only 6. Despite the fact that this group showed a highly significant prevalence of FRT, this result needs to be validated with a much larger cohort. The group of patients with more advanced levels of DR also comprised a relatively smaller number of diabetic subjects. As a result, we may have been underpowered to identify smaller differences in TAS or FRT between some categories. Moreover, it is important to note that this study did not evaluate for the presence or extent of macular ischemia and did not quantify the density of the retinal capillary plexuses by means of OCT angiography. Such an analysis would have strengthened the study by elucidating a direct correlation between flow deficits (ischemia) of the retinal plexuses and the presence of FRT.

In conclusion, this study has identified a significant association of FRT with both ocular and systemic diseases. FRT occurred at all stages of DR and was increasingly prevalent according to the severity of DR and negatively correlated with VA. Further, FRT was positively correlated with diabetes duration, serum creatinine, and glycosylated hemoglobin and negatively correlated with age and eGFR with statistical significance. OCT identification of FRT may provide a surrogate biomarker of retinal and systemic disease.
